# Deubiquitinase OTUD6A Regulates Innate Immune Response via Targeting UBC13

**DOI:** 10.3390/v15081761

**Published:** 2023-08-18

**Authors:** Zhiwei Li, Guanwen Li, Yunfei Li, Yujie Luo, Yuhan Jiang, Ziyu Zhang, Ziyi Zhou, Shengde Liu, Chen Wu, Fuping You

**Affiliations:** 1 College of Life Sciences, Hebei University, Baoding 071002, China; lzw970112@163.com (Z.L.); jyh000818@163.com (Y.J.); zxc4518@163.com (Z.Z.); 18330088099@163.com (Z.Z.); 2 College of Life Sciences, South China Agricultural University, Guangzhou 510642, China; leelearning@163.com; 3 Department of Systems Biomedicine, Institute of Systems Biomedicine, School of Basic Medical Sciences, Peking University Health Science Center, Beijing 100191, China; 1711110048@bjmu.edu.cn (Y.L.); yujieluo@pku.edu.cn (Y.L.); 4 Department of Gastrointestinal Oncology, Key Laboratory of Carcinogenesis and Translational Research, Peking University Cancer Hospital and Institute, Beijing 100142, China

**Keywords:** OTUD6A, IFNβ, UBC13, NF-κB, innate immunity

## Abstract

OTUD6A is a deubiquitinase that plays crucial roles in various human diseases. However, the precise regulatory mechanism of OTUD6A remains unclear. In this study, we found that OTUD6A significantly inhibited the production of type I interferon. Consistently, peritoneal macrophages and bone marrow-derived macrophages from *Otud6a−/−* mice produced more type I interferon after virus infection compared to cells from WT mice. *Otud6a−/−^−^* mice also exhibited increased resistance to lethal HSV-1 and VSV infections, as well as LPS attacks due to decreased inflammatory responses. Mechanistically, mass spectrometry results revealed that UBC13 was an OTUD6A-interacting protein, and the interaction was significantly enhanced after HSV-1 stimulation. Taken together, our findings suggest that OTUD6A plays a crucial role in the innate immune response and may serve as a potential therapeutic target for infectious disease.

## 1. Introduction

The ubiquitin–proteasome pathway (UPP) is the most broadly effective mechanism for modulating a broad range of cellular physiology, including antigen presentation [1,2], homeostasis [3,4], circadian rhythm [5,6], signal transduction [7,8], protein trafficking [9,10], cell differentiation [11,12], immune defense [13,14], and inflammation [15]. The Ubiquitin–Proteasome System (UPS) is composed of ubiquitin (Ub), ubiquitin-activating enzyme (E1), ubiquitin-binding enzyme (E2), ubiquitin-ligase (E3) [16,17,18], 26S proteasome [19], deubiquitinating enzymes (DUBs) [20,21,22], and target proteins. The E1 → E2 → E3 enzymatic cascade covalently attaches the ubiquitin C-terminus to target-specific proteins. This cascade is initiated by E1 and forms a thioester bond between the carboxyl group of the terminal glycine residue of ubiquitin in an ATP-dependent manner [23]. The activated ubiquitin is then transferred to the Cys residue of the E2 enzyme via transesterification, and E2 transfers the ubiquitin to the substrate protein via E3. Ultimately, the 26S proteasome recognizes and degrades the labeled Ub-protein conjugates [24,25], removing most short-lived and aberrant peptides and proteins from eukaryotic cells.

Ubiquitination is involved in regulating signal transduction in antiviral immune responses by precisely activating downstream proteins through pattern recognition receptor signaling, which initiates the innate immune response [26]. Ubiquitylation is a reversible covalent modification that regulates the stability, activity, and localization of target proteins. Deubiquitylating enzymes (DUBs), also known as deubiquitinases, are proteases that remove ubiquitin conjugates from diverse substrate proteins [27]. The human genome contains sixteen ovarian tumor domain-containing 1 (OTU) family members, making them the second largest DUB subfamily; they have emerged as regulators of important signal cascades [28,29].

OTUD1 functions as a metastasis suppressive factor by repressing the metastasis of breast cancer through the deubiquitination of SMAD7 [30]. Additionally, OTUD1 downregulates the induction of type I IFN by cleaving the viral infection-induced atypical K6-linked ubiquitination of IRF3 [31]. A study has shown that OTUB1, a Lys48-specific deubiquitinating enzyme, preferentially binds to the Ubc13~Ub thiolester, which is regulated by free ubiquitin. Free ubiquitin can bind to a second site in OTUB1 and increase the affinity with UBC13, preventing ubiquitin transfer and suppressing the accumulation of polyubiquitin [32,33,34]. Another study has shown that OTUD3, an acetylation-dependent deubiquitinase, shuts down the innate antiviral immune response by directly hydrolyzing lysine 63 (Lys63)-linked polyubiquitination of MAVS [14].

OTUD6A is a member of the OTU domain family DUBs. Previous studies have shown that OTUD6A regulates cell proliferation and tumorigenesis. For instance, OTUD6A deubiquitinates and stabilizes Drp1 to regulate mitochondrial morphology in colon cancer, and it plays a critical role in promoting tumor cell resistance to chemoradiotherapy by deubiquitinating and stabilizing TopBP1 [35,36]. OTUD6A specifically promotes prostate tumorigenesis by stabilizing Brg1, AR, and c-Myc through the removal of Brg1, AR, and c-Myc polyubiquitination, respectively [37,38]. Ubiquitination-dependent processes are critical for producing type I interferon in the antiviral signaling pathway [39,40]. However, whether OTUD6A affects the innate immune response remains unknown.

In this study, we used a library of DUB expression plasmids to screen for DUBs that specifically regulate classical natural immune signaling pathways and found that overexpression of OTUD6A suppressed type I interferon expression. It was found that OTUD6A interacts with UBC13 and not only participates in the regulation of innate immunity but also regulates the inflammatory response. In conclusion, our findings suggest that OTUD6A plays a dual role in regulating innate immunity and inflammatory responses and could provide a potential therapeutic target for related immune diseases.

## 2. Materials and Methods

### 2.1. Cell Culture

HEK293T cells, Hela cells, iBMDM cells, primary bone-marrow-derived macrophage (BMDM) cells, and peritoneal macrophage (PM) cells were cultured in DMEM medium supplemented with 10% FBS (Gibco, Rockville, MD, USA) and 100 U/mL penicillin and 100 mg/mL streptomycin at 37 °C under 5% CO_2_ atmosphere. HEK293T cells, Hela cells, and iBMDM cells were maintained in our laboratory. For the isolation of mouse primary peritoneal macrophages (PMs), cells were collected from the lavage of the peritoneal cavity from mice that were pre-stimulated with thioglycollate (TG) for 3 days. For BMDMs, bone marrow cells were isolated from femurs and tibiae of 8-to-12-week-old *Otud6a−/−* mice and WT mice. Cells were cultured with 20 ng/mL recombinant murine GM-CSF (315-03; Peprotech) in a 10 cm dish for 7 days before experiments.

### 2.2. Reagents and Antibodies

Polyethylenimine (PEI) (764582, Sigma-Aldrich, St. Louis, MO, USA) and jetPRIME (114-15, Polyplus, Strasbourg, France) were used for transfection. DSS (Cat# D8906) was purchased from Sigma-Aldrich. Listeria monocytogenes were provided by the laboratory of Zengfan Jiang, Peking University. The antibodies used in this study were as follows: Anti-OTUD6A (Proteintech, Rosemont, IL, USA, Cat# 24486-1-AP); Anti-NF-κB P65 (CST, Danvers, MA, USA, Cat# 8242); Anti-p-p65 (Ser536) (93H1) (CST, Cat# 3033); Anti-IκBα (CST, Cat# 9242); Anti-p-IκBα (CST, Cat# 5209); Goat anti-mouse IgG, HRP conjugate (Proteintech, Cat# SA00001-1); and Goat anti-rabbit IgG, HRP conjugate (Proteintech, Cat# SA00001-2).

### 2.3. Plasmid Construction

The plasmids used in this study were prepared using standard molecular biology techniques, and the coding sequences were completely verified. Ifnβ promoter luciferase reporter plasmids (IFN-β–luc) and mammalian expression plasmids for human STING, MAVS, TBK1, IRF3, TRIF, and RIG-1 were previously described [41]. All truncations deletions and mutants were constructed using standard molecular biology techniques. Each truncation, deletion, and mutant was confirmed by sequencing.

### 2.4. Luciferase Reporter Assay

293T cells were seeded onto 24-well plates and transfected with the luciferase reporter plasmid, along with the specified expression plasmids or an empty vector (VEC). After 24 h, luciferase activity was measured using the Dual-Luciferase Reporter Assay System (Promega, Madison, WI, USA) following the manufacturers’ instructions.

### 2.5. RNA Extraction and Real-Time PCR (qRT-PCR)

Total RNA from cells, blood, and tissues was extracted using Trizol reagent (Invitrogen, Waltham, MA, USA) and then reverse transcribed into cDNA using HiScript II Q RT SuperMix (Vazyme, Nanjing, China). Quantitative Real-time PCR was performed using the ChamQ Universal SYBR qPCR Master Mix (Vazyme, Nanjing, China) on a 7500 Fast real-time PCR machine (Applied Biosystems, Waltham, MA, USA). The qRT-PCR Data were analyzed using the Livak method (2^−ΔΔCt^) using GraphPad Prism Version 8.0 (GraphPad Software, La Jolla, CA, USA). The data are presented as relative abundance normalized to GAPDH mRNA levels.

Primer sequences are listed as follows:

hIfnβ-F: 5′-AGGACAGGATGAACTTTGAC-3′

hIfnβ-R: 5′-TGATAGACATTAGCCAGGAG-3′

hGapdh-F: 5′- GAAGGTGAAGGTCGGAGTC-3′

hGapdh-R: 5′- GAAGATGGTGATGGGATTTC-3′

mIfnβ-F: 5′-TCCGAGCAGAGATCTTCAGGAA-3′

mIfnβ-R: 5′-TGCAACCACCACTCATTCTGAG-3′

mGapdh-F: 5′- TCACCACCATGGAGAAGGC-3′

mGapdh-R: 5′- GCTAAGCAGTTGGTGGTGCA-3′

mIfit1-F: 5′- CAAGGCAGGTTTCTGAGGAGT-3′

mIfit1-R: 5′- ACCATCAGCATTCTCTCCCAT-3′

mIfit2-F:5′- AGTACAACGAGTAAGGAGTCACT-3′

mIfit2-R: 5′-AGGCCAGTATGTTGCACATGG-3′

mCxcl2-F: 5′-GGGCGGTCAAAAAGTTTGC-3′

mCxcl2-R: 5′-GTTAGCCTTGCCTTTGTTCAGTATC-3′

mCxcl10-F: 5′-CCAAGTGCTGCCGTCATTTTC-3′

mCxcl10-R: 5′-GGCTCGCAGGGATGATTTCAA-3′

mIfna1-F: 5′-GCCTTGACACTCCTGGTACAAATGAG-3′

mIfna1-R: 5′-CAGCACATTGGCAGAGGAAGACAG-3′

mIfna2-F: 5′-CTCTGTGCTTTCCTCGTGATGC-3′

mIfna2-R: 5′-CCTCCTCATCTGTGCCAGGACC-3′

mIfna4-F: 5′-CCTGTGTGATGCAGGAACC-3′

mIfna4-R: 5′-TCACCTCCCAGGCACAGA-3′

mIfna5′-F: 5′-TTTGGATTCCCACAGGAGAAGGTGG-3′

mIfna5′-R: 5′-TCATTGAGCTGCTGATGGACTT-3′

mIL1b-F: 5′-CAACCAACAAGTGATATTCTCCATG-3′

mIL1b-R: 5′-GATCCACACTCTCCAGCTGCA-3′

mIL6-F: 5′-TCTGCAAGAGACTTCCATCCAGTTGC-3′

mIL6-R: 5′-AGCCTCCGACTTGTGAAGTGGT-3′

mISG15′-F: 5′-TGAGGTCTTTCTGACGCAGACTGT-3′

mISG15′-R: 5′-TCAGGCGCAAATGCTTGATCACTG-3′

mTNFα-F: 5′-ACAGAAAGCATGATCCGCG-3′

mTNFα-R: 5′-GCCCCCCATCTTTTGGG-3′

mIL12b-F: 5′-TGGGAGTACCCTGACTCCTG-3′

mIL12b-R: 5′-AGGAACGCACCTTTCTGGTT-3′

VSV-G-F: 5′-CAAGTCAAAATGCCCAAGAGTCACA-3′

VSV-G-R: 5′-TTTCCTTGCATTGTTCTACAGATGG-3′

HSV-1-F: 5′-GCAGAAGGTCTCCGGTAAT-3′

HSV-1-R: 5′-CGCTACGATACGACACCAA-3′

### 2.6. RNA Sequencing (RNA-Seq)

The whole RNA of cells with specific treatment was purified using RNeasy Mini Kit (QIAGEN NO. 74104). The transcriptome library for sequencing was generated using VAHTSTM mRNA-seq v2 Library Prep Kit for Illumina (Vazyme Biotech, Nanjing, China) according to the manufacturer’s recommendations. Following library preparation, the libraries were sequenced on the Illumina Hiseq X Ten platform using (2 × 150 bp) paired-end module. The raw images were transformed into raw reads by base calling using CASAVA software (https://support.illumina.com.cn/sequencing/sequencing_software/casava.html, accessed on 14 January 2017). Subsequently, the raw reads in a Fastq format were processed using in-house PERL scripts. Clean reads were obtained by removing reads with adapters, reads with more than 5% unknown bases, and low-quality reads (defined as reads with more than 50% low-quality bases, where a low-quality base has a sequencing quality below 10). Additionally, the Q20 and Q30 scores, as well as the GC content of the clean data, were calculated (Vazyme Biotech, Nanjing, China). The original data of the RNA-seq were uploaded to the GEO DataSets.

### 2.7. Virus Infection in Cell and Mice

Wild-type C57BL/6 mice were obtained from the Department of Laboratory Animal Science of Peking University Health Science Center, Beijing. The mice were bred and housed under specific pathogen-free conditions. For all in vivo experiments, age- and sex-matched mice were used unless otherwise specified. Six-week-old mice were infected with HSV-1 virus (5 × 10^7^ plaque-forming unit (PFU) virus) or VSV (3 × 10^8^ PFU per mouse). At the indicated times after infection, the mice were sacrificed, and blood samples were collected. The expression of cell cytokine and viral genes were analyzed using RT-qPCR.

All animal care and mouse experiments were conducted in accordance with ethical guidelines and approved by the Animal Care and Use Committee of Peking University. The project was assigned the license number LA2016239. *Otud6a−/−* mice were purchased from Shanghai Model Organisms Center, Shanghai.

### 2.8. Mass Spectrometry

To identify proteins that potentially interact with OTUD6A, purified Flag-OTUD6A samples from 293T and Hela cells were subjected to mass spectrometry analysis. The purification was performed using Flag antibody prior to the mass spectrometry experiments.

### 2.9. Coimmunoprecipitation and Immunoblot Assays

HEK293 cells and Hela cells were seeded on 10 cm dishes (1 × 10^7^ cells/dish). The cells were then transfected with a total of 10 μg of Flag-OTUD6A expression plasmids or an empty plasmid. After 24 h of transfection, the cells were lysed in lysis buffer (0.5% Triton X-100, 20 mM HEPES (pH 7.4), 150 mM NaCl, 12.5 mM β-glycerolphosphate, 1.5 mM MgCl_2_, 2 mM EGTA, 10 mM NaF, 1 mM Na_3_VO_4_, 2 mM DTT) supplemented with protease inhibitors. The lysates were centrifuged, and the supernatant was incubated with anti-Flag antibodies at 4 °C overnight. The following day, prewashed protein A/G beads (Pierce, Waltham, MA, USA) were added to the lysates and incubated at 4 °C for 4 h. The beads were then washed four times with cold PBS and eluted with SDS sample buffer containing DTT by boiling for 10 min in preparation for western blotting.

### 2.10. Statistical Analysis

Differences between experimental and control groups were evaluated using Student *t*-test when comparing two groups of data or one-way ANOVA analysis when comparing more than two groups of data. Statistical analysis and data visualization were performed using GraphPad Prism 8.0 software. The data were presented as mean ± standard deviation (SD). The p values were as follows: ns *p* > 0.05, * *p* < 0.05, ** *p* < 0.01, *** *p* < 0.001, and **** *p* < 0.0001.

## 3. Results

### 3.1. OTUD6A Overexpression Inhibits the Production of Type I IFN

**Figure 1:**
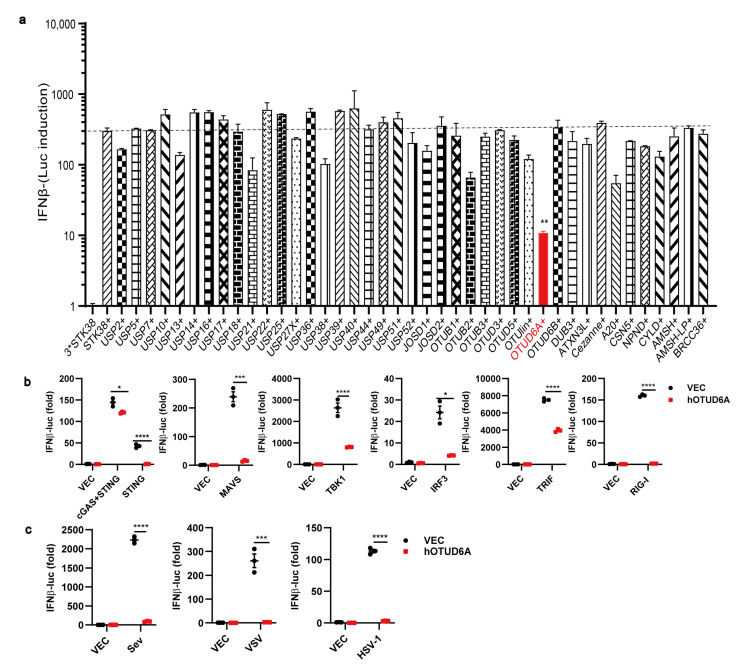
Overexpression of OTUD6A restricts innate immune signaling. (**a**) HEK293 cells were seeded onto 24-well cell culture plates and transfected with 50 ng of Ifnβ-Luci and 20 ng of Actin-Luci reporter gene plasmids, along with 100 ng of Flag-cGAS and 100 ng of Flag-STING expression plasmids in each well. After 12 h, the cells were co-transfected with 42 DUBs expression plasmids. Twenty-four hours later, the activation of the reporter gene was measured using a single-well microplate reader. (**b**) HEK293 cells were transfected with IFNβ-Luci and Actin-Luci reporter gene plasmids, along with Flag-OTUD6A expression plasmids. The cells were then co-transfected with STING, MAVS, TBK1, IRF3, TRIF, and RIG-1 expression plasmids, respectively. The control group was transfected with an empty vector plasmid. After 24 h, the biological activity of dual luciferase was assessed using a single-well microplate reader. (**c**) HEK293 cells were transfected with Flag-OTUD6A expression plasmid or an empty vector plasmid and then infected with SeV, VSV, and HSV-1 viruses after 24 h. After 24 h of infection, the biological activity of dual luciferase was measured using a single-well microplate reader. (**a**–**c**) Student’s *t*-test; Significance levels are noted as * *p* < 0.05, ** *p* < 0.01, *** *p* < 0.001 and **** *p* < 0.0001. All values are presented as mean ± SEM.

To investigate the impact of the OTUD family on the innate immune response, we constructed all the human OTUD expression plasmids. In addition to transfecting deubiquitinases, we co-transfected STING and cGAS in 293T cells to activate the expression of *Ifnβ*. Luciferase reporter assays demonstrated that among the OTUDs tested, OTUD6A exhibited the most significant inhibition of *Ifnβ* expression (Figure 1a), indicating its potential as a negative regulator of the cGAS-STING signaling pathway. To further assess its impact on other canonical pattern recognition receptors (PRRs) such as RIG-I and innate immunity downstream proteins such as MAVS, TBK1, IRF3, TRIF, and STING, we co-transfected OTUD6A and observed that it significantly inhibited the induction of *Ifnβ* by RIG-I/MAVS, cGAS/STING, TBK1/IRF3, and TRIF (Figure 1b), suggesting its involvement in these signal pathways and role as a component of the innate immune signaling pathway. Additionally, we explored the impact of OTUD6A on the innate immune responses mediated by the DNA virus HSV-1 and RNA viruses VSV and SeV. As hypothesized, overexpression of OTUD6A led to significant inhibition of *Ifnβ* expression following infection with any of these viruses (Figure 1c), thus confirming its negative regulatory role in the expression of *Ifnβ*.

### 3.2. OTUD6A Deficiency Enhances Antiviral Innate Immunity In Vitro

**Figure 2:**
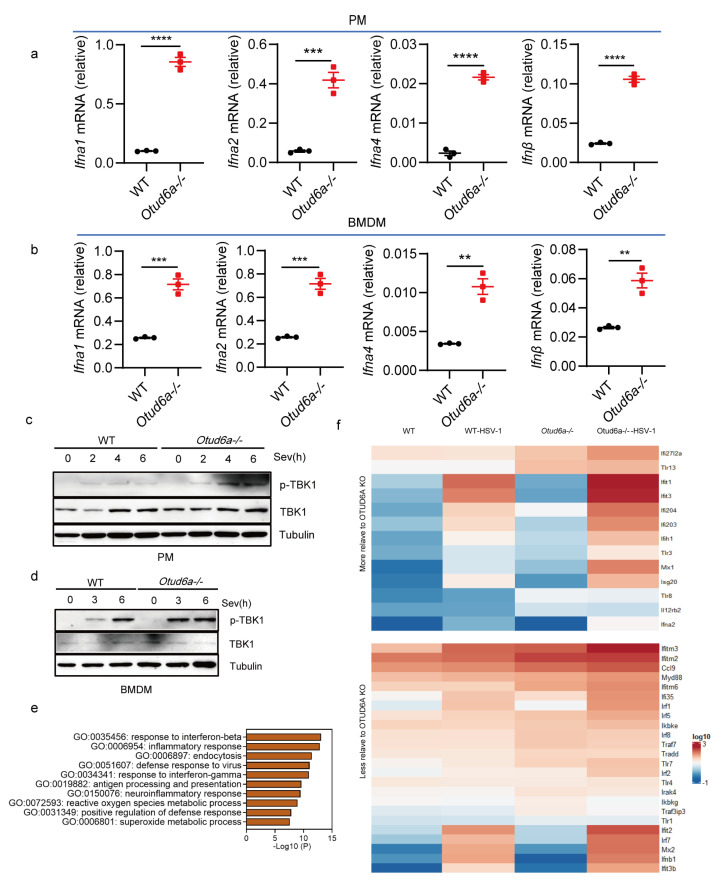
OTUD6A deficiency enhances antiviral innate immunity in vitro. (**a**,**b**) The mRNA expression levels of *Ifna1*, *Ifna2*, *Ifna4*, and *Ifnβ* were measured by RT-qPCR in *Otud6a−/−* mouse-derived peritoneal macrophages (PMs) and bone marrow-derived macrophages (BMDMs) infected with Sev (0.1 MOI) for 8 h, compared to wild-type (WT) cells. (**c**,**d**) Changes in TBK1 phosphorylation were detected by Western Blot when mouse peritoneal macrophages and bone marrow-derived macrophages were infected with Sev virus (0.1 MOI) at different times, respectively. (**e**) RNA extracted from *Otud6a−/−* and wild-type iBMDM cells, 6 h after HSV-1 infection, was used for whole transcriptome sequencing. Differentially expressed genes were identified and subjected to gene ontology (GO) analysis. (**f**) The heatmap was generated to represent the normalized expression levels of viral infection-induced genes, as indicated. (**a**,**b**) Student’s *t*-test; Significance levels are noted as ** *p* < 0.01, *** *p* < 0.001, and **** *p* < 0.0001. All values are presented as mean ± SEM.

To gain further insights into the function of OTUD6A in the innate immune responses, we generated *Otud6a−/−* mice and infected peritoneal macrophages (Figure 2a and Figure S1a,b) and BMDMs (Figure 2b) isolated from both wild-type and *Otud6a−/−* mice with Sev. We then assessed the mRNA levels of type I interferon using RT-qPCR. Our results showed that the expression of *Ifna1*, *Ifna2*, *Ifna4*, and *Ifnβ* was significantly increased in *Otud6a−/−* peritoneal macrophages and BMDMs, which was not due to OTUD6A knockout by default (Figure S1d), confirming that OTUD6A played a critical role as a negative regulator of the innate immune response. At the same time, the expression of *Il6* decreased, and the expression of *Tnf-α* and *Il12* did not change in *Otud6a−/−* PMs (Figure S1c). Western data showed that the phosphorylation level of TBK1 in *Otud6a−/−* PMs or BMDMs (Figure 2c,d) is higher than that in WT after Sev infection. We also performed whole-transcriptome sequencing on wild-type and *Otud6a−/−* iBMDM cells infected with HSV-1 and analyzed the top antiviral response-related terms from GO biological processes, which indicated a strong correlation between innate immunity signal pathways and OTUD6A (Figure 2e). Moreover, the expression of some antiviral-related genes was significantly higher in cells from *Otud6a−/−* mice after HSV-1 infection (Figure 2f). These findings suggested that OTUD6A was an important negative regulator of innate immunity.

### 3.3. OTUD6A Deficiency Enhances Antiviral Innate Immunity In Vivo

**Figure 3:**
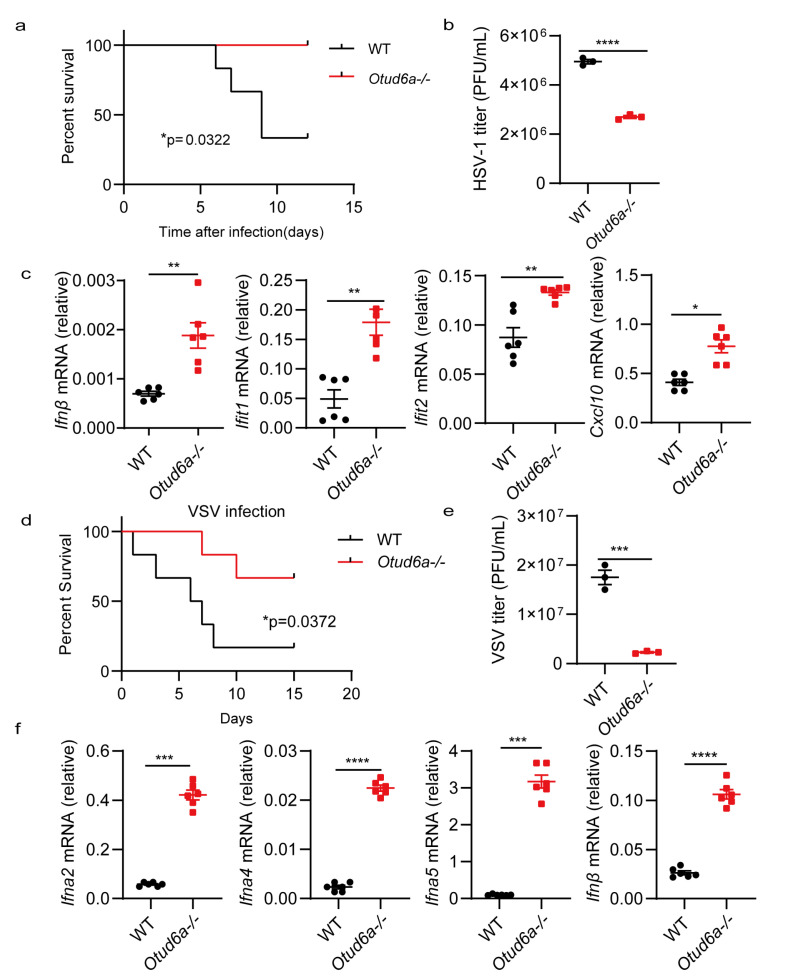
OTUD6A deficiency enhances antiviral innate immunity in vivo. (**a**) Wild-type (WT) mice and *Otud6a−/−* mice (n = 6) were subjected to tail vein injection of HSV-1 (5 × 10^7^ PFU per mouse), and the mice were monitored for survival. (**b**) On the fifth day after the HSV-1 virus infection, brain tissue was extracted from the mice to measure virus titers using plaque assays. (**c**) One day after HSV-1 infection, total mRNA was extracted from the blood, and the mRNA levels of *Ifnβ*, *Ifit1*, *Ifit2,* and *Cxcl10* were measured using RT-qPCR. (**d**) WT mice and *Otud6a−/−* mice (n = 6) were tail vein injected with VSV (1 × 10^8^ PFU per mouse), and mouse survival was monitored. (**e**) On the fifth day after the VSV virus infection, brain tissue was extracted from the mice to determine virus titers using plaque assays. (**f**) One day after VSV infection, total mRNA was extracted from the blood, and the mRNA levels of *Ifna2*, *Ifna4*, *Ifna5*, and *Ifnβ* were measured using RT-qPCR. (**a**,**d**) Log-rank test; (**b**,**c**,**e**,**f**) Student’s *t*-test; significance levels are noted as * *p* < 0.05, ** *p* < 0.01, *** *p* < 0.001, and **** *p* < 0.0001. All values are presented as mean ± SEM.

In vitro experiments demonstrated that the absence of OTUD6A enhanced innate immunity. To investigate the function of OTUD6A in vivo, we established an acute infection model by injecting HSV-1 into mice. Our results showed that *Otud6a−/−* mice had increased virus resistance and a higher survival rate than wild-type mice (Figure 3a). We also extracted mouse brain tissue on the fifth day after the virus infection and detected the virus load. The plaque results indicated that the virus titer in the brain of *Otud6a−/−* mice was lower than that in wild-type mice (Figure 3b). Furthermore, we collected blood from mice infected with the virus for different days and found that *Otud6a−/−* mice produced more type I interferon and stronger antiviral-related factors (*Ifnβ*, *Ifit1*, *Ifit2*, and *Cxcl10*) compared to wild-type mice (Figure 3c). We next investigated if OTUD6A had a similar effect on RNA viruses. To this end, we injected WT and *Otud6a−/−* mice with vesicular stomatitis virus (VSV) and observed that *Otud6a−/−* mice had a higher survival rate and lower viral RNA levels (Figure 3d,e and Figure S2). Additionally, the expression of antiviral-related factors, such as *Ifna2*, *Ifna4*, *Ifna5*, and *Ifnβ*, was significantly higher in the blood of *Otud6a−/−* mice than in wild-type mice (Figure 3f). These findings demonstrated that OTUD6A played a critical role in facilitating innate immune responses to viral infections.

### 3.4. OTUD6A Deficiency Attenuates the Inflammatory Response In Vitro and In Vivo

**Figure 4:**
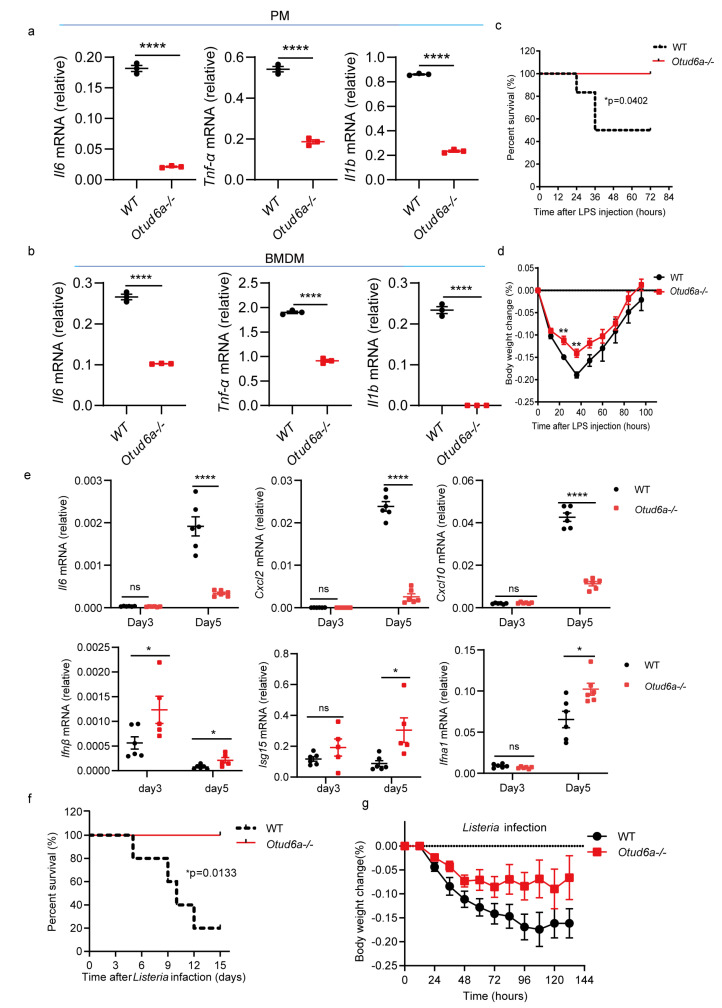
OTUD6A deficiency attenuates the inflammatory response. (**a**,**b**) Peritoneal macrophages and bone marrow-derived macrophages were isolated from wild-type and *Otud6a−/−* mice and stimulated with 1 μg/mL LPS for 6 h. Total mRNA was extracted, and the mRNA levels of *Il6*, *Tnf-α*, and *Il1b* were measured using RT-qPCR. (**c**,**d**) Wild-type and *Otud6a−/−* mice were intraperitoneally injected with LPS (8.74 μg/g). The mice were observed daily for mortality and changes in body weight in each group. (**e**) RT-qPCR analysis was performed to measure the mRNA levels of *Il6*, *Cxcl2*, *Cxcl10*, *Ifnβ*, *Isg15,* and *Ifna1* in the serum (*n* = 6) obtained from wild-type or *Otud6a−/−* mice intraperitoneally injected with LPS for 3 and 5 days. (**f**,**g**) Wild-type mice and *Otud6a−/−* mice (*n* = 5) were injected intraperitoneally with *Listeria monocytogenes* (1 × 10^7^ CFU per mouse) and observed for mortality and weight change in each group. (**a**,**b**,**e**) Student’s *t*-test; (**c**,**f**) Log-rank test; significance levels are noted as * *p* < 0.05, ** *p* < 0.01 and **** *p* < 0.0001, and ns is not significant. All values are presented as mean ± SEM.

A recent study showed that OTUD6A can promote inflammation response during tumorigenesis [42]. We isolated primary peritoneal macrophages and bone marrow-derived macrophages from WT and *Otud6a−/−* mice and stimulated them with LPS. RT-qPCR showed that compared with wild-type cells, the expression of inflammatory factors, such as *Il1b*, *Il6*, and *Tnf-α*, decreased in both *Otud6a−/−* PMC and *Otud6a−/−* BMDM (Figure 4a,b and Figure S3a,b). Our results also showed that OTUD6A facilitated inflammation response, which is consistent with previous work.

To explore the role of OTUD6A in vivo, we established an acute infection model by infecting mice with LPS. The results showed that compared with WT mice, *Otud6a−/−* mice had higher survival rates (Figure 4c) and less body weight loss (Figure 4d). Moreover, *Otud6a−/−* mice had higher serum *Ifnβ, Isg15, Ifna1* and lower *Il6*, *Cxcl2* and *Cxcl10* levels than WT mice (Figure 4e). Consistently, *Otud6a−/−* mice have much higher survival rates compared with WT mice after 2% DSS treatment (Figure S3c). *Otud6a−/−* mice also have higher survival rates and less body weight loss after *listeria* infection (Figure 4c,f,g). These results showed that OTUD6A promoted the induction of proinflammatory cytokines after LPS treatment and played a critical role in regulating inflammation in vivo.

### 3.5. OTUD6A Participates in the Regulation of NF-κB Mediated Inflammation Signaling Pathway via UBC13

**Figure 5:**
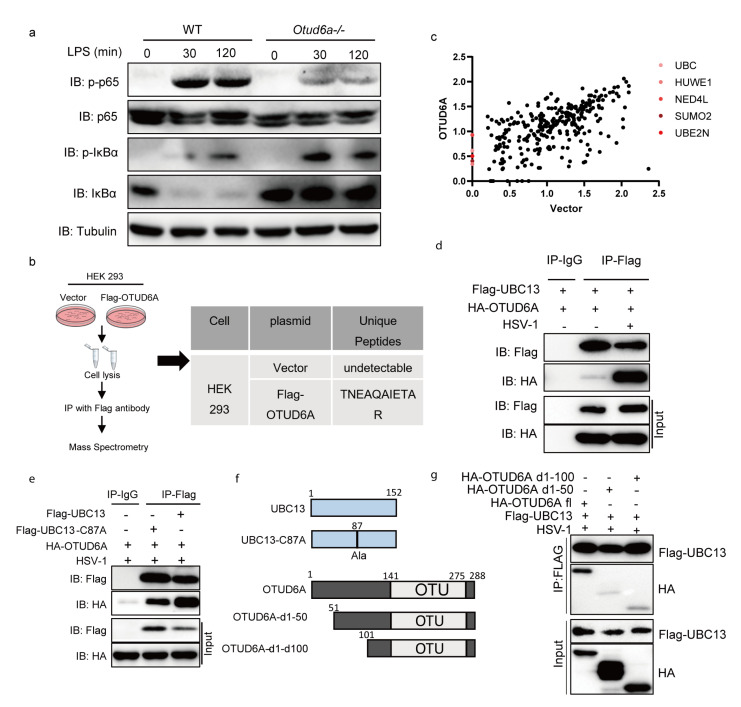
OTUD6A participates in the regulation of NF-κB mediated inflammation signaling pathway via UBC13. (**a**) Macrophages derived from the bone marrow of wild-type and *Otud6a−/−* mice were induced and stimulated with 1 μg/mL LPS for 30 min and 120 min. Total proteins were extracted for an immunoblotting experiment to detect the protein levels of endogenous p65, p-p65, IκBα, and p-IκBα. (**b**) FLAG-M2 beads were used to enrich proteins in HEK293 cells, re Ubc13 was identified as a potential interaction protein for OTUD6A through mass spectrometry. (**c**) Log_2_(score) of Flag-vector and Flag-OTUD6A MS. Flag-OTUD6A MS specific ubiquitin-relative proteins were highlighted. (**d**) Flag-UBC13 and HA-OTUD6A were simultaneously overexpressed in HEK293 cells. After 24 h, the cells were infected with HSV-1. Co-immunoprecipitation experiments were performed using Flag antibody and IgG antibody after 6 h, respectively. (**e**) The cysteine residue at position 87 of UBC13 was mutated to alanine to obtain the Flag-UBC13-C87A mutant plasmid. HA-OTUD6A and Flag-Ubc13 or Flag-UBC13 C87A plasmids were overexpressed in HEK293 cells for 24 h, followed by HSV infection. Co-immunoprecipitation experiments were performed using Flag antibody and IgG antibody after 6 h, respectively. (**f**) The pattern of UBC13 point mutations and OTUD6A truncation mutations is shown. (**g**) OTUD6A with 50 or 100 amino acids missing from the N-terminal (HA-OTUD6A d1-50, HA-OTUD6A d1-100) and full-length OTUD6A (HA-OTUD6A fl) were transfected into HEK293 cells with UBC13 for 24 h. The cells were then infected with HSV for 6 h before co-immunoprecipitation (with anti-FLAG) and immunoblotting analysis using the indicated antibodies.

OTUD6A is a critical component of antiviral innate immunity and inflammatory signaling pathways, but its specific mechanism remains unclear. To further investigate the mechanism, we induced mouse peritoneal macrophages and infected them with LPS. Western blotting results showed that *Otud6a−/−* macrophages exhibited reduced levels of phosphorylated p65 compared to WT cells, while the phosphorylated and total IκBα levels increased (Figure 5a). This suggested that the deletion of OTUD6A inhibited the degradation process of phosphorylated IκBα, indicating that the NF-κB pathway is not efficiently activated. Recent research has shown that OTUD6A directly binds to the NACHT domain of the NLRP3 inflammasome and selectively cleaves K48-linked polyubiquitin chains to enhance the stability of NLRP3 [42]. In conclusion, OTUD6A could promote inflammation.

To identify potential target proteins interacting with OTUD6A, we overexpressed Flag-OTUD6A or empty vector in HEK 293 cells and enriched them with a Flag antibody for mass spectrometry analysis. Our results showed that the ubiquitin-conjugating enzyme UBE2N (UBC13) was a specific interacting protein of OTUD6A (Figure 5c). UBC13 is a critical E2-conjugating enzyme in the regulation of immunity and inflammation, which can prevent the conversion of regulatory T cells into effector-like T cells [43], promote K63-Linked polyubiquitination of NLRP3 [44], and confer processivity to TRAF6 ubiquitin ligase activity [45]. Immunoprecipitation analysis confirmed that the interaction between OTUD6A and UBC13 was present in the resting state and significantly enhanced after HSV-1 stimulation (Figure 5d). We also investigated the role of Cys87, a key enzyme activity site of UBC13, on their interaction and found that the loss of this site did not affect their interaction (Figure 5e). Furthermore, truncation of the first 50 or 100 amino acids at the N-terminal of OTUD6A significantly reduced its interaction with UBC13, suggesting that OTUD6A interacted with UBC13 through its N-terminal (Figure 5f,g). These findings suggested that OTUD6A may rely on UBC13 to participate in the regulation of NF-κB-mediated inflammation signaling pathways.

## 4. Discussion

The balance between immunity and inflammation responses is dynamic under steady-state conditions, but can quickly shift in response to pathogen invasion [46,47]. The family of deubiquitinases plays an essential role in regulating this balance, as different deubiquitinases may either up- or down-regulate innate immunity or inflammation response [48,49,50]. In this study, we demonstrated that OTUD6A can attenuate the anti-virus innate immunity response and promote inflammation response.

Our findings demonstrate that overexpression of OTUD6A led to the downregulation of *Ifnβ* expression induced by RIG-I/MAVS, cGAS/STING, TBK1/IRF3, or TRIF overexpression. These results indicate that OTUD6A had a broad inhibitory effect on the production pathway of *Ifnβ*. It is worth noting that OTUB1 and OTUB2 negatively regulate the expression of *Ifnβ* induced by the VISA-mediated pathway, but not the TBK1-mediated pathway [51]. The downregulation of *Ifnβ* expression during VSV, SeV, or HSV-1 infection was also observed upon overexpression of OTUD6A. In vitro experiments demonstrated that OTUD6A-deficient BMDMs and PMCs expressed more interferons when infected with SeV compared to WT. Furthermore, in vivo experiments revealed that *Otud6a−/−* mice expressed more interferons when infected with VSV or HSV-1 and were more resistant to death than WT mice. NGS analysis showed that *Otud6a−/−* iBMDM expressed more interferons when infected with HSV-1 compared to WT.

In vitro LPS treatment in *Otud6a−/−* BMDMs and PMCs showed that *Otud6a−/−* mice were more resistant to death induced by LPS or DSS compared to WT. These findings are consistent with the work of Xin Liu et al. [42], which showed that OTUD6A directly bound to the NACHT domain of the NLRP3 inflammasome and selectively cleaved K48-linked polyubiquitin chains from NLRP3 at K430 and K689 to enhance the stability of NLRP3, leading to increased *Il1β* levels and inflammation [42]. In vivo DSS treatment in *Otud6a−/−* mice also confirmed that OTUD6A promoted the induction of proinflammatory cytokines.

To elucidate the mechanism by which OTUD6A participates in the immune response, we employed immunoprecipitation (IP) and mass spectrometry (MS) to enrich and identify potential interacting proteins of OTUD6A. Our results revealed that OTUD6A could interact with UBC13, and further verification experiments demonstrated that this interaction was partly dependent on the N-terminal of OTUD6A. UBC13 is a well-known E2 ubiquitin-conjugating enzyme and immune receptor, which has an important role in the process of tagging target proteins with lysine 63-linked polyubiquitin chains that are essential for the activation of NF-κB [52,53]. Recent research has shown that dendritic cell-specific deletion of OTUB1 impaired cytokine production after *T. gondii* infection by increasing the stability of UBC13. Conversely, OTUD1 physically interacted with receptor-interacting serine/threonine-protein kinase 1 (RIPK1) and selectively cleaved K63-linked polyubiquitin chains from RIPK1 to inhibit the recruitment of NF-κB essential modulator (NEMO), thereby inhibiting the inflammatory response [53]. This controversy suggests the diverse roles of deubiquitinases in regulating immune and inflammatory responses, indicating that they have redundant and opposite effects on inflammation response pathways. Furthermore, we found that *Otud6a−/−* PMC exhibited a reduced level of phosphorylated p65 but increased levels of phosphorylated and total IκBα, indicating that OTUD6A promotes the degradation process of pIκBα.

In summary, our findings demonstrated that OTUD6A plays a critical role in regulating both innate immunity and inflammatory response. Our data revealed that OTUD6A can both inhibit innate immunity and promote inflammatory response, highlighting its importance in accelerating the cellular response to deadly virus or bacterial infections. These results provided new insights for the treatment of infectious and inflammatory diseases, emphasizing the potential therapeutic value of targeting OTUD6A to modulate immune and inflammatory responses.

## Data Availability

We cannot provide RNA-seq data due to unpublished results. Other data can be provided upon request.

## Supplementary Materials

The following supporting information can be downloaded at: https://www.mdpi.com/article/10.3390/v15081761/s1, Figure S1: RT-qPCR of OTUD6A deficient PMs after viruses infection in vitro. Figure S2: OTUD6A deficiency increases innate immunity in vivo and in vitro. Figure S3: OTUD6A deficiency attenuates inflammatory response in vitro and in vivo.
