# Supplementary material for: Deubiquitinase OTUD6A Regulates Innate Immune Response via Targeting UBC13
Source: Viruses. 2023 Aug 18;15(8):1761. doi: 10.3390/v15081761 (PMC10458163; doi:10.3390/v15081761)
Supplement: Supplementary file 1 [file viruses-15-01761-s001.zip › viruses-2485686-supplementary.pdf]

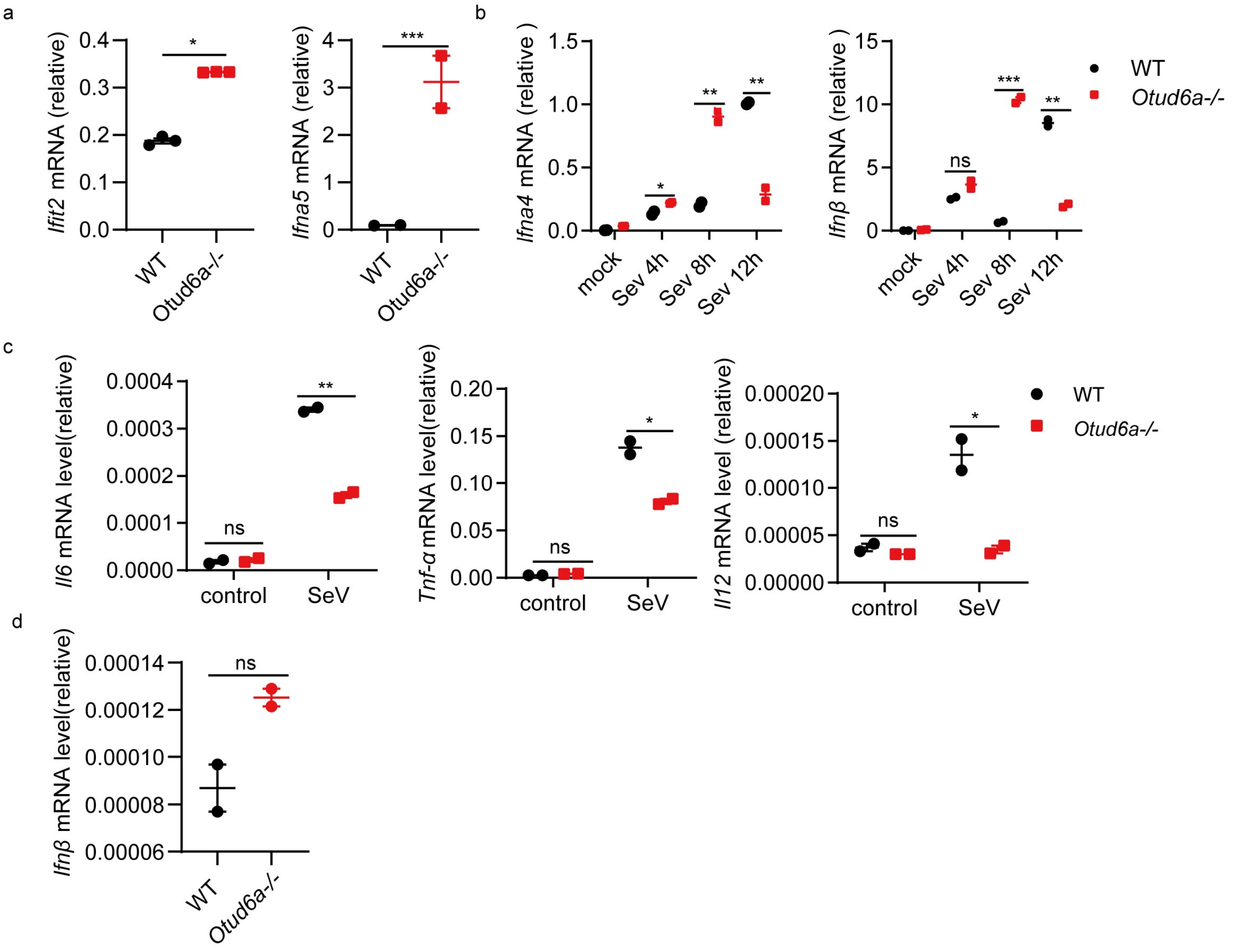

Figure S1. RT-qPCR of OTUD6A deficient PMs after viruses infection in vitro. (**a**) mRNA expression of *Ifit2* and *Ifna5* in WT or *Otud6a*−/−mouse-derived PMs infected with Sev (0.1 MOI) for 8h were assessed by RT-qPCR. (**b**) Peritoneal macrophages were obtained from wild-type and *Otud6a*−/− mice, and the cells were infected with Sev (0.1 MOI) virus for 0, 4, 8, and 12 h. Cellular *Ifna4* and *Ifnβ* mRNA expression levels were detected by qPCR. (**c**) Peritoneal macrophages were obtained from wild-type and *Otud6a*−/− mice, and the cells were infected with Sev (0.1 MOI) virus for 8 h, Cellular *Il6*,*Tnf-α* and *Il2* mRNA expression levels were detected by qPCR. (**d**) qPCR was performed to detect the expression of *Ifnβ* in WT and *Otud6a*−/− PM cells when the cells were in the absence of bacterial infection. (**a**-**d**) Student's t-test; Significance levels are noted as *$p < 0.05$, **$p < 0.01$, ***$p < 0.001$ and ****$p < 0.0001$; ns, not significant. All values are presented as mean ± SEM.

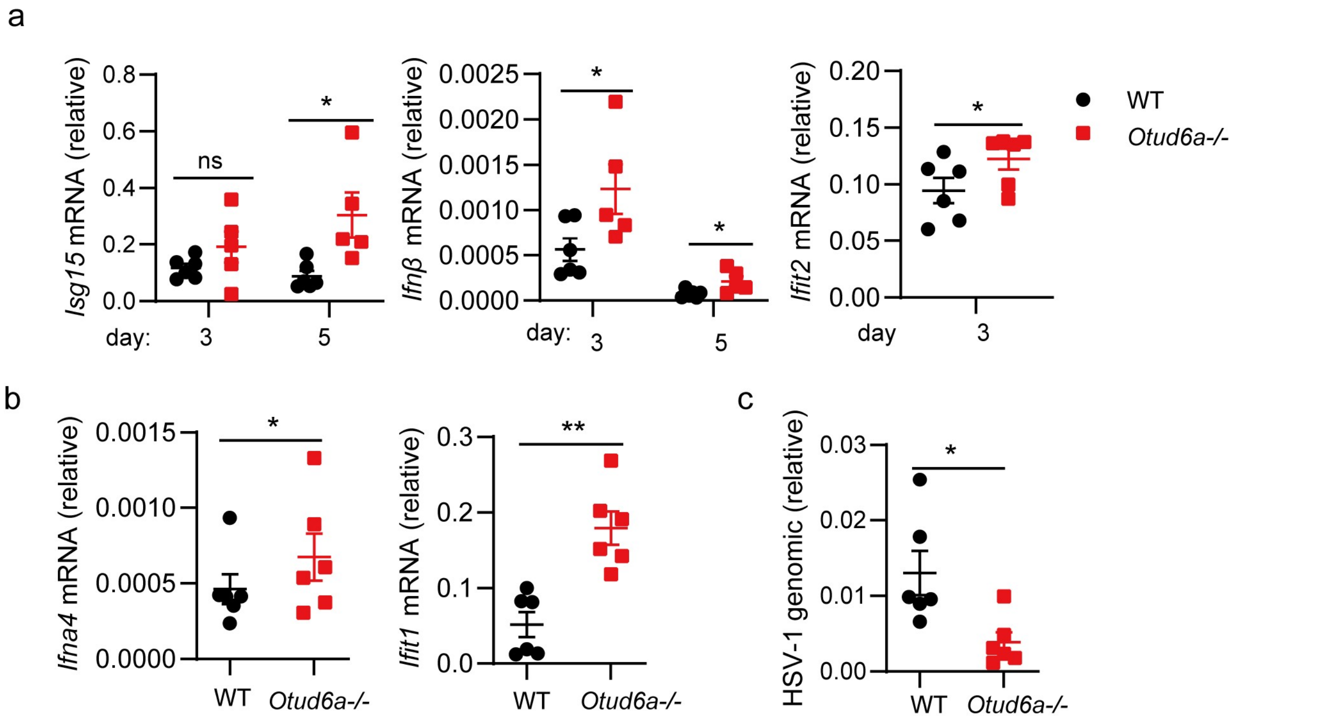

Figure S2. OTUD6A deficiency increases innate immunity in vivo and in vitro. (**a**) On the third and fifth day of HSV-1 infection in mice, total mRNA was extracted from blood and the mRNA levels of *Isg15*, *Ifnβ* and *Ifit2* were measured by qPCR, respectively. (**b**) On the third day of VSV infection in mice, total mRNA was extracted from blood and the mRNA levels of *Ifna4* and *Ifit1* were measured by qPCR. (**c**) On the third day of HSV-1 infection in mice, total virus load was measured by RT-qPCR. (**a**,**b**) Student's t-test; Significance levels are noted as $*p < 0.05$, $**p < 0.01$; All values are presented as mean ± SEM.

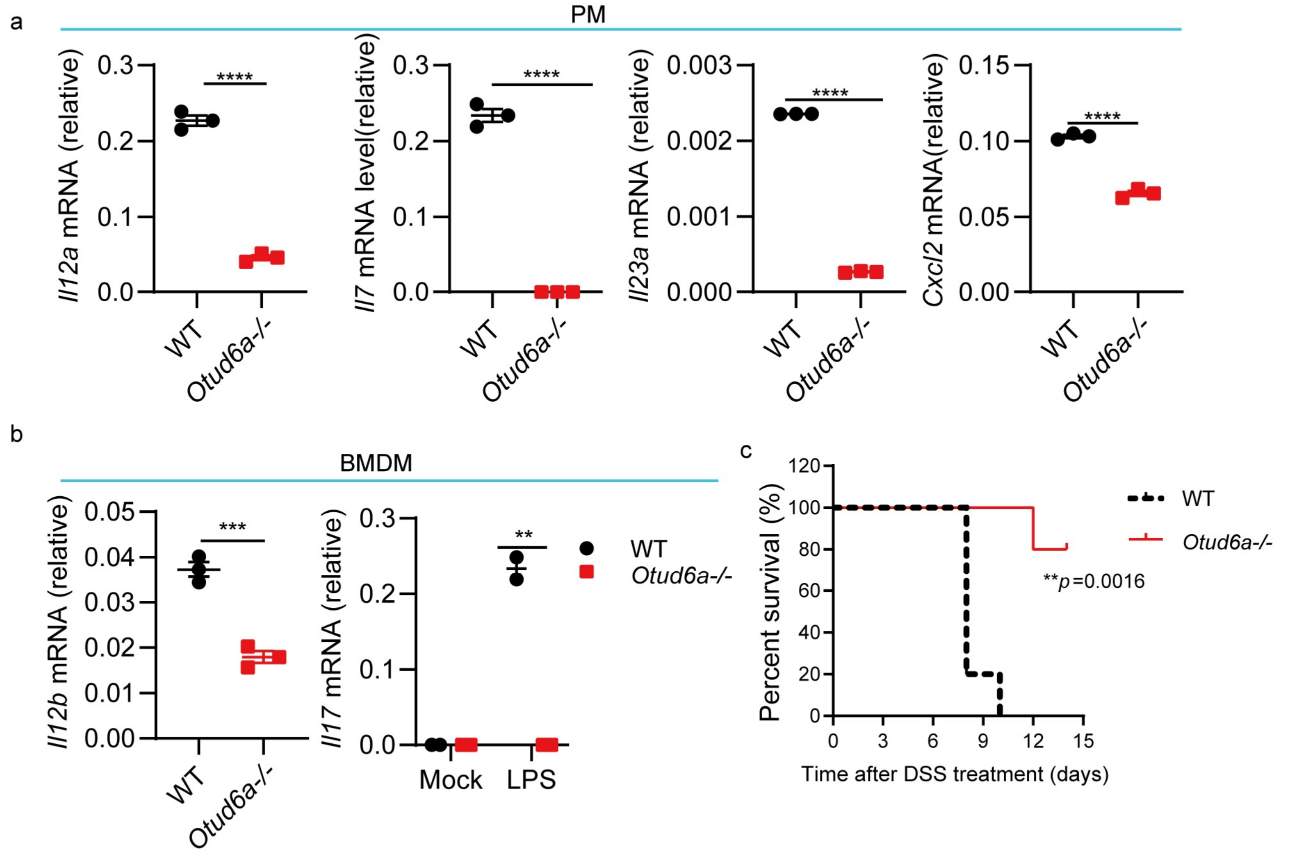

Figure S3. OTUD6A deficiency attenuates inflammatory response in vitro and in vivo. (**a**) Peritoneal macrophages were obtained from wild-type and *Otud6a*−/−mice, stimulated with 1 μg/mL LPS for 6 h. Total mRNA was extracted and mRNA levels of *Il12a*, *Il7*, *Il23a* and *Ccl2* genes were measured by RT-qPCR. (**b**) Bone marrow-derived macrophages were obtained from wild-type and *Otud6a*−/− mice, stimulated with 1 μg/mL LPS for 6 h. Total mRNA was extracted and mRNA levels of *Il12b* and *Il17* genes were measured by RT-qPCR. (**c**) Wild-type mice and *Otud6a*−/−mice (n = 5) were fed with 2% DSS water for one week and then replaced with normal water, and the mortality of each group was recorded. (**a**,**b**) Student's t-test; (**c**) Log-rank test; Significance levels are noted as $^{**}p < 0.01$, $^{***}p < 0.001$ and $^{****}p < 0.0001$; ns, not significant. All values are presented as mean ± SEM.
